# Glucose Metabolism: The Metabolic Signature of Tumor Associated Macrophage

**DOI:** 10.3389/fimmu.2021.702580

**Published:** 2021-06-29

**Authors:** Qi Zhang, Junli Wang, Dipesh Kumar Yadav, Xueli Bai, Tingbo Liang

**Affiliations:** ^1^ Department of Hepatobiliary and Pancreatic Surgery, The First Affiliated Hospital, Zhejiang University School of Medicine, Hangzhou, China; ^2^ Zhejiang Provincial Key Laboratory of Pancreatic Disease, The First Affiliated Hospital, Zhejiang University School of Medicine, Hangzhou, China; ^3^ Zhejiang Clinical Research Center of Hepatobiliary and Pancreatic Diseases, Hangzhou, China; ^4^ The Innovation Center for the Study of Pancreatic Diseases of Zhejiang Province, Hangzhou, China; ^5^ Zhejiang University Cancer Center, Hangzhou, China

**Keywords:** macrophage, glucose metabolism, polarization, cancer, therapy

## Abstract

Macrophages exist in most tissues of the body, where they perform various functions at the same time equilibrating with other cells to maintain immune responses in numerous diseases including cancer. Recently, emerging investigations revealed that metabolism profiles control macrophage phenotypes and functions, and in turn, polarization can trigger metabolic shifts in macrophages. Those findings implicate a special role of metabolism in tumor-associated macrophages (TAMs) because of the sophisticated microenvironment in cancer. Glucose is the major energy source of cells, especially for TAMs. However, the complicated association between TAMs and their glucose metabolism is still unclearly illustrated. Here, we review the recent advances in macrophage and glucose metabolism within the tumor microenvironment, and the significant transformations that occur in TAMs during the tumor progression. Additionally, we have also outlined the potential implications for macrophage-based therapies in cancer targeting TAMs.

## Introduction

Cancer is a major public health burden worldwide, with a significantly high incidence of mortality. The environment around the tumor is called as tumor microenvironment (TME), which assists cancer cells in growth and progression (1). Over the last few years, TME has extensively been studied for the effective treatment of cancer. Though TME has diverse tumor-infiltrating immune cells like the T-cells, regulatory T-cells (Treg), myeloid-derived suppressor cells (MDSC), tumor-associated neutrophils, dendritic cells, and tumor-associated macrophages (TAMs), macrophages are the most abundant (2). A large number of studies suggest that TAMs serve as a key promoter of metastasis in cancer, by releasing extracellular signals, growth factors, proteolytic enzymes, and inhibitory proteins for T cells (3). Thus, targeting TAMs to prevent tumor progression and metastasis has been a hot spot in current cancer research.

Traditionally, macrophages are the large phagocytes that pose various forms in tissues throughout the body (e.g., Kupffer cells in the liver, alveolar macrophages in the lungs, microglia in the cerebrum) and typically play an important role in homeostatic and immune responses during the disease process (4, 5). Moreover, macrophages are highly plastic and can modify their properties subsequently according to the microenvironment (6). Inactive macrophages (M0) typically represent undifferentiated cells and can reprogram themselves into polarized cells when exposed to certain stimuli. Depending on the cell surface markers, cytokines release, and metabolic signatures, macrophages are conventionally classified into two subtypes, i.e. classically activated pro-inflammatory M1 macrophages, and alternatively activated anti-inflammatory M2 macrophages (5, 7–9).

In recent years, increasing evidence has put forward that TAMs can unanimously adopt distinct metabolic signatures to execute proper effector functions required for the TME (10–13). It has been traditionally assumed that cancer cells primarily metabolize glucose *via* glycolysis to produce sufficient energy and other key metabolites necessary for survival (Warburg effect) (14–17), which essentially perplexes the metabolic profiles of immune cells especially TAMs (18). However, a fresh study astonishingly revealed that TAMs are the main consumer of glucose in cancers rather than cancer cells themselves (19). Yet, how glucose metabolism influences TAMs functions in cancer and vice versa are still obscure. Consequently, the complex correlation between glucose metabolism and TAMs in TME is worthy to investigate adequately. In this review, we have focused on the modifications that consistently occur in glucose metabolism and TAMs in TME, and the potential implications for macrophage-based therapies in cancer.

## Glucose Metabolism Pathways

Glucose traditionally serves as the primary source of energy for supporting the normal functions of the cells including macrophages. After being transported across the plasma membrane, glucose is principally metabolized through three pathways, i.e. glycolysis, pentose phosphate pathway (PPP), and Krebs or Tricarboxylic Acid (TCA) cycle (20, 21). Glycolysis is a metabolic pathway typically takes place in the cytosol, which breaks down glucose into pyruvate in aerobic environment and lactate in anaerobic settings and produces adenosine triphosphate (ATP). Pyruvate produced from aerobic glycolysis further enters the Krebs cycle and is oxidized through a series of reactions called oxidative phosphorylation (OXPHOS) to produce more ATPs. On the other hand, glycolysis also supplies glucose-6-phosphate to the PPP, provoking the production of nicotinamide adenine dinucleotide phosphate (NADPH) and ribose-5-phosphate. Though glycolysis possesses a lower capacity for ATP generation than OXPHOS, (only two ATP per molecule of glucose), it is a more rapid source of energy for macrophages and other cells and contributes metabolic intermediates for biosynthetic pathways to support the synthesis of ribose, amino acids, and fatty acids that are crucial for metabolic adaptation (22, 23). Apart from the above-mentioned three glucose metabolism pathways, glucose can further be metabolized *via* the hexosamine biosynthesis pathway (HBP) (2–5%) and eventually leading to the generation of a donor molecule uridine diphosphate N-acetylglucosamine (UDP-GlcNAc) (24–26).

Macrophages preferentially attach the surface of glucose transporter 1 (GLUT1) to meet their energy requirements (27). Under normal conditions, naïve M0 macrophages get energy by efficiently employing OXPHOS (28). Whereas, polarized macrophages (M1 and M2) rely more on their characteristic metabolic signatures for energy prerequisite within the tissue microenvironment (23).

## Glucose Metabolism and The M1 Macrophages

Traditional pro-inflammatory cytokine such as interferon γ (IFN-γ), tumor necrosis factor α (TNF-α), and lipopolysaccharide (LPS) stimulates M0 macrophages to differentiate into classical M1 phenotype (29–31). M1 macrophages exhibit profound inflammatory cytokines secretion (including IL-1β, IL-6, IL-23, TNF-α) and precise antigen presentation (**Table 1**). To uphold dramatic pro-inflammatory functions, M1 macrophages trigger energy expenditure by the magnified aerobic glycolysis and PPP in conjunction with decreased OXPHOS and fatty acid oxidation (FAO). Glycolysis and PPP are fundamental for macrophage functional adjustments and preventing the body from harmful events within an exigent time.

**Table 1:** The complexity between macrophage phenotypes and glucose metabolism.

	**M1 macrophage**	**M2 macrophage**	**Tumor-associated Macrophage**
Activation stimuli	IFN-γ, LPS, TNF-α	IL-4, IL-13	Tumor microenvironment, such as hypoxia, adenosine
Inflammatory cytokines secretion	IL-1β, IL-6, IL-12, IL-23, TNF-α	IL-1, IL-6, IL-10, TGF-β	Both, mainly anti-inflammatory cytokines
Marker expression	CD68, CD86, CD80, MHC-II, INOS, TLR-4	CD163, CD206, MHC-II, CXCR1, CXCR2, TLR1, TLR8	Both M1 & M2 markers, mainly immunosuppressive molecules
Chemokine secretion	CXCL3, CXCL5, CCL2, CCL3, CCL4, CCL5, CCL8-11	CCL17, CCL18, CCL22, CCL24	CCL1, CCL5, CCL10
Antigen presentation	Yes	No	Yes
Glucose metabolism pattern	Glycolysis, PPP, HBP	OXPHOS, FAO, HBP	OXPHOS & FAO, with increased glycolysis, PPP, HBP
Glucose metabolism enzymes	HK, PFKFB3, PKM2, PDK1	PDK1, CARKL, PFKFB1	both
Signaling pathways	HIF-1α, STAT1, STAT5, IRF3, IRF5, NF-κb	mTORC2, IRF4, STAT3, STAT6	AKT/mTOR, HIF-1α, NF-κb
Functions	Pro-inflammatory, tissue damage	Anti-inflammatory, phagocytosis; tumor formation and progression	M2a, M2b, M2c, M2d and others subtypes; promoting tumor progression; immune suppression; immune scape

In parallel, glycolytic enzymes are found to have remarkable alternations within the LPS microenvironment (32). Traditionally, glycolysis is mainly regulated by three major enzymes: hexokinase (HK), phosphofructokinase 1 (PFK1), and pyruvate kinase (PK), which catalyze irreversible steps in this process (21). Under LPS stimulation, HK acts as the glucose sensor and mediates the phosphorylation of glucose for subsequent utilization, crucially contributing to the pro-inflammatory cytokine secretion in M1 macrophages (33). Recently, an inducible form of PFK1, 6-phosphofructo-2-kinase/fructose-2,6-bisphosphatase 3 (PFKFB3) stepped into research (34, 35). Once PFKFB3 is stimulated with IFN-γ/LPS, it further induces progressive production of fructose 2,6-bisphosphate, and thus, promotes overall glycolysis flux in M1 macrophages to meet its energy demand (36). On the other hand, M1 macrophages significantly upregulate the key metabolic regulator, an isoform 2 of the pyruvate kinase (PKM2) under LPS activation to bind IL-1β promotor region concerning increased inflammatory response (37, 38).

Besides, overexpression of GLUT1 in M1 macrophages promotes glucose metabolism and metabolites production in the PPP, striking a complex pro-inflammatory signature (39). It has been found that long-term glucose exposure reduces the phagocytic ability of M1 macrophages, probably because of impaired glycolytic capacity (40). Interestingly, the constitutive expression of sedoheptulose kinase (CARKL), a carbohydrate kinase-like protein that is involved in the conversion of sedoheptulose into sedoheptulose-7-phosphate, decreases the glycolytic flux of glucose and results in defective M1 polarization (41) These findings portray an interlaced network that pro-inflammatory molecules stimulate glucose metabolism in macrophages. Conversely, glucose uptake in macrophages supervises pro-inflammatory phenotype. The pro-inflammatory environment and increased glucose levels might guide each other in a self-perpetuating cycle, among which hypoxia-induced factor 1 alpha (HIF-1α) (37, 42–44) plays an essential role.

Previously, HBP was identified to promote inflammation in macrophages that associated with O-linked β-N-acetylglucosamine (O-GlcNAc) signaling (45, 46). Nevertheless, a study surprisingly observed a decreased HBP activity and protein O-GlcNAcylation in LPS-stimulated macrophages. Subsequently, they proved that the O-GlcNAcylation of the receptor-interacting serine/threonine-protein kinase 3 contributed to an unexpected inhibitory effect (47). Indeed, Yang et al. observed a similar immunosuppressive role of O-GlcNAc signaling in macrophage activation. Macrophages presented suppressed O-GlcNAc signaling during M1 polarization even though the increased glucose uptake. Therefore, macrophage O-GlcNAc signaling is an important regulator of integrating glucose metabolism and inflammatory response. Taken together, those results indicated that metabolic changes are not just the result of the inflammatory response, but rather a critical modulator of the entire process.

## Glucose Metabolism and the M2 Macrophages

Alternatively activated M2 macrophages are primarily induced by IL-4 and IL-13 that are secreted from innate and adaptive immune cells, and are characterized by an anti-inflammatory profile mainly IL-10 and transforming growth factor-beta (TGF-β) (8, 29–31). In contrast to M1 macrophages, M2 macrophages preferentially utilize FAO and OXPHOS to execute cellular behaviors and activities (**Table 1**) (48–50). Although some evidence demonstrated that FAO is typical for M2 polarization, researchers believe that M2 macrophages retain the same dependence on glycolysis and exhibit modest glucose consumption (51, 52). Glucose can fuel fatty acid synthesis to support increased FAO in M2 macrophages, linking glycolysis, fatty acid synthesis, and FAO.

An integrative analysis demonstrated that glucose oxidation, but not that of fatty acids, is necessary for the early differentiation of M2 macrophages and PDK-1 plays an ineffable role in this conversion (53). Glucose uptake was increased over time in macrophages when stimulated by IL-4. This observation pioneeringly spiked interest of glycolysis in M2 macrophages (54). Another point as recognized, CARKL is upregulated in M2 macrophages, which can lead to the production of ribose-5P, enhancing the nonoxidative steps of PPP (41). Moreover, a selective expression of the glycolytic enzyme 6-phosphofructo 2-kinase B1 (PFKFB1), was consistently found in M2 macrophage, it can catabolize fructose-2,6-bisphosphate more efficiently than PFKFB3.

Alluringly, it was found that blocking glycolysis with 2-deoxyglucose (2-DG) diminished the IL-4-induced expression of the M2 phenotype, and the mTORC2 signaling upstream of IRF4 expression played a critical role (54, 55). Interestingly, similar results were acquired from macrophages cultured in a glucose-free medium (55). Depletion of glucose or substitution of glucose with galactose remarkably suppresses glycolysis but does not affect OXPHOS and M2 macrophages activation (51). This phenomenon indicates that glycolysis is not mandatory for M2 activation if OXPHOS is intact, but becomes necessary if OXPHOS is compromised (56). At the same time, HBP was also found dispensable for anti-inflammatory M2-like polarization (57). Thus, glucose looks like energy support for OXPHOS in M2 macrophages, probably triggering a spurt mitochondrial respiratory activity.

## Glucose Metabolism Signature of Tumor-Associated Macrophages

As stated earlier, TAMs constitute the largest population of immune cells within the tumor, and are immunosuppressive in nature during tumor progression. Upregulation of the expression of ectonucleoside triphosphate diphosphohydrolase 1 (ENTPD also known as CD39), 5’-nucleotidase Ecto (NT5E also known as CD73) (58, 59), or programmed cell death ligand 1 (PDL-1) (60) were comprehensively detected in TAMs. As cancer cells themselves are typically dependent on glucose, they consume most glucose from the surrounding microenvironment and administrate glycolysis to supply rapidly growing energy requirements. Consequently, TAMs domestically shift toward OXPHOS and FAO metabolism and exhibit functions primarily similar to M2 macrophages in a poor glucose TME to maintain their immunosuppressive roles (61, 62). Wenes et al. recently revealed that in hypoxic conditions of solid tumors, TAMs promoted neoangiogenesis and tumor metastasis by shift towards oxidative metabolism with decreased glycolysis through activation of mTOR signaling pathways (63). In the meanwhile, results showed that enhanced glucose flux through the HBP propelled cancer progression by boosting O-GlcNAcylation in TAMs (64).

However, slightly distinction of environment stimulus can elicit substantially different macrophage phenotypes and metabolism profiles (65, 66). Even though given the same stimuli, macrophages can display differential responsiveness. Considering the complexity of the TME, the plasticity and adaptability of macrophages, it should be noted that such a defined 2D spectrum of M1–M2 polarization adopted from *in-vitro* experiments may not properly map the metabolism signatures of macrophage *in-vivo*, it has to be considered as an extremely dynamic and mixed 3D spectrum. More recently researches revealed that TAMs actually have higher glucose uptake (67) and a high level of glycolytic metabolism similar to M1 macrophages to support their cytokine profiles and functions. Proteomic analyses revealed that glycolytic enzymes including hexokinase 2 are upregulated in macrophages stimulated by tumor extract solution from breast cancer patients (68), consistent with the findings in pancreatic ductal adenocarcinoma (PDAC) (69) and non-medullary thyroid carcinoma (70). Simultaneously, lactic acid released by glycolytic cancer cells into the TME also upregulates HIF-1α expression in TAMs responsible for increased glycolysis and M2-like state (71, 72). Additionally, *in-vivo*, macrophages are capable of repolarization from M2 to dichotomous M1 phenotype, they can co-express both M1 and M2 polarization hallmarks following tumor progression (56). We recently identified a subtype of pro-inflammatory M2-type (CD206+IL-1β+) TAMs characterized as stable mitochondrial respiration, enhanced glycolysis, and elevated O-GlcNAcylation protein levels in hepatocellular carcinoma. This novel subtype of macrophages shares similar cell markers and cellular metabolism with classic M2-like phenotype while playing a pro-inflammatory M1-like function (73). Other researchers too have found such enhanced glycolysis in TAMs and have recognized more subtypes of TAMs in cancer like CD68+ TAM in non-small cell lung cancer (NSCLC) (74), CD169+ macrophages in PADC (75, 76), CD163+ macrophages in epithelial ovarian cancer (77), and PD-1+ macrophages in primary mouse and human cancer (78). Hence, all various phenotypes of TAMs can contribute to the tumor progression, depending on the metabolism balance in TME.

By integrating data from the ImmGen project, Schultze et al. proposed a core signature for human and murine macrophages expanding our understanding (79). Correspondingly, Sarukhan et al. discussed the potential underlying mechanisms regulating TAMs specialization (80). These studies allowed us better understand the heterogeneity of TAMs in tumors. Nevertheless, the question that how glucose metabolism influence macrophages’ switch in the tumor microenvironment, involving the recruitment of circulating precursors or the re-education of cells *in situ* still existed. Our group recently identified a novel subtype of CD19+ TAMs in HCC, results showed that glycolysis may be an innate feature that prefers the tumor progression (unpublished data). A recent study also supported that glucose use was modulated by cell-intrinsic programs of cells through mTORC1 signaling in tumor (19). In fact, tumor cells rely more on glucose to support their growth than TAMs, such nutrient competition between tumor cells and immune cells apparently are adverse for the ready proliferation of tumor cells. Additionally, how macrophages glucose metabolism affects other immune cells in tumor is incompletely explored. Hence, more profound work is required to develop the underlying process.

Delightfully, advances in technology for single-cell RNA sequencing (81, 82) and high-dimensional cytometry by fluorescence or mass cytometry (cytometry by time of flight (CyTOF)) (83) significantly promoted the high-dimensional single-cell analyses. In the past few years, numerous profound and novel views of metabolic flux and TAMs have been stated (84, 85). In further study, the complete and elaborate description of TAMs subpopulations landscape remains to establish to explain the macrophages evolution and glucose metabolism.

## Target Glucose Metabolism In Macrophage for Cancer Therapy

**Figure 1:**
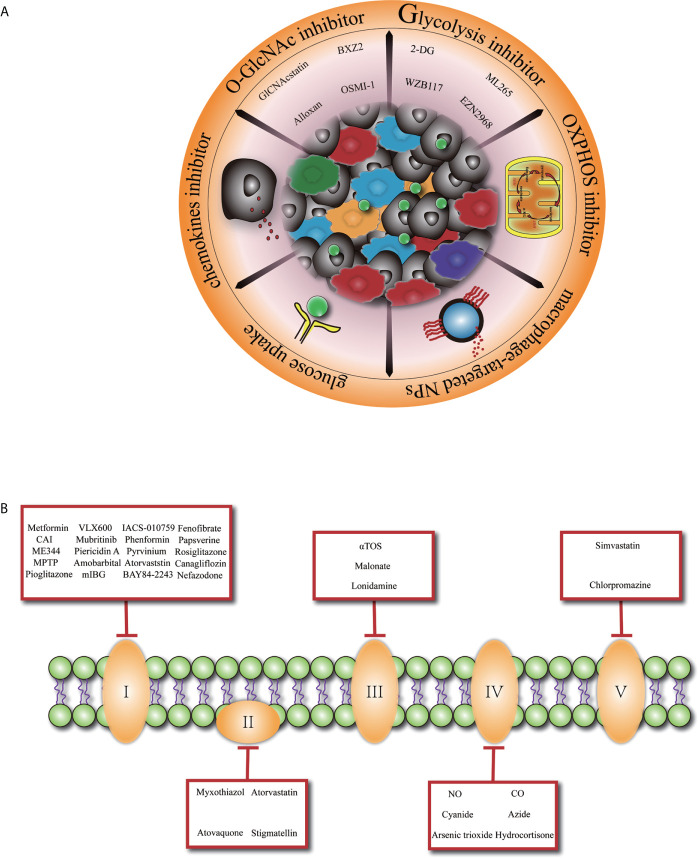
Glucose metabolism basis macrophage-targeted therapy for cancer. **(A)** Overview of promising cancer therapy based on glucose metabolism characteristic in tumor-associated macrophage. 2-DG, 2-deoxyglucose. **(B)** Specific presentation of OXPHOS (oxidative phosphorylation) inhibitors involved mitochondrial complex I, II, III, IV, V. CAI, carboxyamidotriazole; MPTP, 1-methyl 4-phenyl 1,2,3,6 tetrahydropyridine; mIBG2, meta-iodobenzylguanidine; aTOS, a-tocopheryl succinate; NO, nitric oxide; CO, carbon monoxide.

Given the important role of TAMs in promoting tumor development and the complex landscape of the macrophages which are heterogeneously evolved under the selective pressure of TME, manipulating macrophages tentatively may serve as a promising approach for controlling tumor progression (**Figure 1A**). Previously, TAMs-targeted antitumor strategies were mainly based on the inhibition of macrophages recruitment (86, 87) or depletion of M2-like TAMs. However, a recent study discovered that interruption of C–C motif chemokine ligand 2 (CCL2) inhibition was associated with increased cancer cell mobility and neovascularization, leading to accelerated metastasis and cancer death (88). Furthermore, far-ranging macrophage depletion could bring outside effects, such as other immunosuppressive cells’ compensation (89–91).

Since macrophages glucose metabolism is inextricably connected to its functionality, metabolic reprogramming of M2-like TAMs toward an anti-tumoral phenotype at the same time rupture cancer cell metabolism might be an elegant way. In the context of a profound relationship between OXPHOS and the differentiation of M2 macrophages especially in TAMs, inhibiting OXPHOS pathway (**Figure 1B**) has been explored as a promising approach to promote TAMs transition to M1 macrophages (92). Blocking the expression of succinate dehydrogenase complex flavoprotein subunit A (SDHA) and oxidative phosphorylation activities of macrophages with dimethyl malonate treatment exhibited markedly delayed tumor growth (93). Similarly, FAO inhibitors are developed to achieve the phenotypic transition of macrophages and inhibit tumor development. Furthermore, researchers have revealed that acriflavine (ACF), a heteroaromatic dye with an antibacterial and antiviral effect, shifted macrophage polarization to an M1-like anti-tumoral phenotype by blocking the HIF-1α pathway and enhancing glucose uptake in PDAC (94). This phenomenon shows that increasing the glucose utilization of TAMs may be a promising direction.

As above mentioned, glycolysis is important in the early differentiation of TAMs, the maintenance of an M2-like profiles also dependent on a high glycolytic flow. Consequently, glycolysis inhibition (with decreased lactate derived from the tumor) of TAMs is certainly hopeful for cancer therapy. Chitin administration significantly decreased anti-inflammatory M2 macrophage polarization and prevented disease progression in a series of mouse models (95). Also, dichloroacetic acid profoundly prevented macrophage migration in a lung tumor xenograft model by inhibiting macrophages glycolysis (42). Several O-GlcNAcylation inhibitors had been proved to inhibit cancer cell growth (96, 97). Nevertheless, specific targeting of one of the metabolic pathways for macrophages is potentially deflective. Proper adjustment of glucose metabolism in macrophages, instead of a simple one-way increase or decrease, presents a potential therapeutic strategy.

In addition, owing to distinct cell populations of the TME share common metabolic profiles and all metabolic pathways are important for normal cells, sustained modifications of core metabolic pathways may have marginally immunological effects that are difficult to predict. Alternatively, the use of prodrugs that are specifically activated macrophages according to the embellishment of glucose metabolism in TME could be considered for future therapy. For instance, esterase-sensitive motif (ESM) inhibitors were prosperously tested as clinical agents targeting macrophages (98). More than that, with the development of nanotechnology, drug delivery systems based on nanoparticles (NPs) have been in the generation of therapeutic agents for several features, they are avirulent and can easily penetrate physiological barriers with a stable consistency. Glucose-based NPs have been used as biocompatible polymers to re-educate TAMs (99). Meanwhile, ^18^F-FDG PET (100–102) has been proposed as a non-invasive strategy to detect glucose uptake and orbit underlying macrophage polarization mechanisms. The application of biological or chemical materials in targeted therapy makes it possible for the natural modulation of macrophage glucose metabolism *in-vivo*, favoring an optimal metabolic balance of macrophages to display functions in TME.

## Conclusion

As previously described, macrophages might respond diversely depending on the heterogeneity in miscellaneous tissue microenvironment and cell subpopulations ongoing changed. Hence, macrophages should be considered as dynamic alternations in the different phases of cancer where they adapt various phenotypes and also metabolic signatures; the enhanced or decreased glucose metabolism of macrophages should also not be taken as favorable or harmful effects for TME. On the other hand, advanced tools such as spatial transcriptomics and multiplex immunohistochemistry need to be developed to dig the association of glucose metabolism and macrophages. Whatever, currently, the most effective strategies to target cancer will have to precisely combine TAM-targeted prodrugs delivery systems with complex cell glucose metabolism pathways and real-time imaging systems in cancer. In summary, depth work is required to probe the macrophage-response specificity, tissue-type sensitivity, and metabolism-pattern availability, especially constricting the gap between research and clinic with the help of precision medicine.

## Author Contributions

QZ and JW wrote the manuscript. DY, XB, and TL revised the manuscript. QZ and JW contributed equally to this work. All authors contributed to the article and approved the submitted version.

## Funding

This work was financially supported by the National Key Research and Development Program (2020YFA0804300), National Natural Science Foundation of China (Nos. 81871320, 82071865) and the Distinguished Young Scholar Foundation of Zhejiang Province, China (LR20H160002).

## Conflict of Interest

The authors declare that the research was conducted in the absence of any commercial or financial relationships that could be construed as a potential conflict of interest.

## References

[1] Joyce JA Fearon DT. T Cell Exclusion, Immune Privilege, and the Tumor Microenvironment. Sci (New York N.Y.) (2015) 348:74–80. 10.1126/science.aaa6204 25838376

[2] Netea-Maier RT Smit JWA Netea MG. Metabolic Changes in Tumor Cells and Tumor-Associated Macrophages: A Mutual Relationship. Cancer Lett (2018) 413:102–9. 10.1016/j.canlet.2017.10.037 29111350

[3] Lin Y Xu J Lan H. Tumor-Associated Macrophages in Tumor Metastasis: Biological Roles and Clinical Therapeutic Applications. J Hematol Oncol (2019) 12:76. 10.1186/s13045-019-0760-3 31300030 PMC6626377

[4] Ovchinnikov DA. Macrophages in the Embryo and Beyond: Much More Than Just Giant Phagocytes. Genesis (2008) 46:447–62. 10.1002/dvg.20417 18781633

[5] Italiani P Boraschi D. New Insights Into Tissue Macrophages: From Their Origin to the Development of Memory. Immune Netw (2015) 15:167–76. 10.4110/in.2015.15.4.167 PMC4553254 26330802

[6] Viola A Munari F Sánchez-Rodríguez R Scolaro T Castegna A. The Metabolic Signature of Macrophage Responses. Front Immunol (2019) 10:1462. 10.3389/fimmu.2019.01462 31333642 PMC6618143

[7] Mills CD Kincaid K Alt JM Heilman MJ Hill AM. M-1/M-2 Macrophages and the Th1/Th2 Paradigm. J Immunol (2000) 164:6166–73. 10.4049/jimmunol.1701141 10843666

[8] Gordon S. Alternative Activation of Macrophages. Nat Rev Immunol (2003) 3:23–35. 10.1038/nri978 12511873

[9] Yao Y Xu XH Jin L. Macrophage Polarization in Physiological and Pathological Pregnancy. Front Immunol (2019) 10:792. 10.3389/fimmu.2019.00792 31037072 PMC6476302

[10] Langston PK Shibata M Horng T. Metabolism Supports Macrophage Activation. Front Immunol (2017) 8:61. 10.3389/fimmu.2017.00061 28197151 PMC5281575

[11] DeNardo DG Ruffell B. Macrophages as Regulators of Tumour Immunity and Immunotherapy. Nat Rev Immunol (2019) 19:369–82. 10.1038/s41577-019-0127-6 PMC7339861 30718830

[12] Vitale I Manic G Coussens LM Kroemer G Galluzzi L. Macrophages And Metabolism in the Tumor Microenvironment. Cell Metab (2019) 30:36–50. 10.1016/j.cmet.2019.06.001 31269428

[13] Su P Su P Su P Ma X Liu L Yang M. Enhanced Lipid Accumulation and Metabolism are Required for the Differentiation and Activation of Tumor-Associated Macrophages. Cancer Res (2020) 80:1438–50. 10.1158/0008-5472.CAN-19-2994 PMC7127942 32015091

[14] Warburg O Wind F Negelein E. The Metabolism OF Tumors IN the Body. J Gen Physiol (1927) 8:519–30. 10.1085/jgp.8.6.519 PMC2140820 19872213

[15] Warburg O. On Respiratory Impairment in Cancer Cells. Sci (New York N.Y.) (1956) 124:269–70. 13351639

[16] Vander Heiden MG Cantley LC Thompson CB. Understanding the Warburg Effect: The Metabolic Requirements of Cell Proliferation. Sci (New York N.Y.) (2009) 324:1029–33. 10.1126/science.1160809 PMC2849637 19460998

[17] Jang M Kim SS Lee J. Cancer Cell Metabolism: Implications for Therapeutic Targets. Exp Mol Med (2013) 45:e45. 10.1038/emm.2013.85 24091747 PMC3809361

[18] Hsu PP Sabatini DM. Cancer Cell Metabolism: Warburg and Beyond. Cell (2008) 134:703–7. 10.1016/j.cell.2008.08.021 18775299

[19] Reinfeld BI Madden MZ Wolf MM Chytil A Bader JE Patterson AR. Cell-Programmed Nutrient Partitioning in the Tumour Microenvironment. Nature (2021) 593:282–8. 10.1038/s41586-021-03442-1 PMC8122068 33828302

[20] O’Neill LA Kishton RJ Rathmell J. A Guide to Immunometabolism for Immunologists. Nat Rev Immunol (2016) 16:553–65. 10.1038/nri.2016.70 PMC5001910 27396447

[21] Werner C Doenst T Schwarzer M. Metabolic Pathways and Cycles. Scientist’s Guide to Cardiac Metab (2016) 39–55. 10.1016/B978-0-12-802394-5.00004-2

[22] Xie N Zhang L Gao W Huang C Huber PE Zhou X. NAD(+) Metabolism: Pathophysiologic Mechanisms and Therapeutic Potential. Signal Transduction Targeted Ther (2020) 5:227. 10.1038/s41392-020-00311-7 PMC7539288 33028824

[23] Liu Y Xu R Gu H Zhang E Qu J Cao W. Metabolic Reprogramming in Macrophage Responses. Biomarker Res (2021) 9:1. 10.1038/s41392-020-00311-7 PMC7786975 33407885

[24] Kreppel LK Blomberg MA Hart GW. Dynamic Glycosylation of Nuclear and Cytosolic Proteins: CLONING and CHARACTERIZATION of A Unique O-GlcNAc Transferase WITH Multiple TETRATRICOPEPTIDE Repeats*. J Biol Chem (1997) 272:9308–15. 10.1074/jbc.272.14.9308 9083067

[25] Love DC Hanover JA. The Hexosamine Signaling Pathway: Deciphering the “O-GlcNAc Code”. Science’s STKE Signal Transduction Knowl Environ (2005) 2005):re13. 10.1126/stke.3122005re13 16317114

[26] Yang X Ongusaha PP Miles PD Havstad JC Zhang F So WV. Phosphoinositide Signalling Links O-GlcNAc Transferase to Insulin Resistance. Nature (2008) 451:964–9. 10.1038/nature06668 18288188

[27] Fukuzumi M Shinomiya H Shimizu Y Ohishi K Utsumi S. Endotoxin-induced Enhancement of Glucose Influx Into Murine Peritoneal Macrophages Via GLUT1. Infect Immun (1996) 64:108–12. 10.1128/iai.64.1.108-112.1996 PMC173734 8557327

[28] Van den Bossche J Baardman J de Winther MP. Metabolic Characterization of Polarized M1 and M2 Bone Marrow-Derived Macrophages Using Real-Time Extracellular Flux Analysis. J Visualized Exp JoVE (2015) 105:53424. 10.3791/53424 PMC4692751 26649578

[29] Murray PJ Allen JE Biswas SK Fisher JA Gilroy DW Goerdt S. Macrophage Activation and Polarization: Nomenclature and Experimental Guidelines. Immunity (2014) 41:14–20. 10.1016/j.immuni.2014.06.008 25035950 PMC4123412

[30] Murray PJ. Macrophage Polarization. Annu Rev Physiol (2017) 79:541–66. 10.1146/annurev-physiol-022516-034339 27813830

[31] Ramond E Jamet A Coureuil M Charbit A. Pivotal Role of Mitochondria in Macrophage Response to Bacterial Pathogens. Front Immunol (2019) 10:2461. 10.3389/fimmu.2019.02461 31708919 PMC6819784

[32] Freemerman AJ Johnson AR Sacks GN Milner JJ Kirk EL Troester MA. Metabolic Reprogramming of Macrophages: Glucose Transporter 1 (GLUT1)-Mediated Glucose Metabolism Drives a Proinflammatory Phenotype. J Biol Chem (2014) 289:7884–96. 10.1074/jbc.M113.522037 PMC3953299 24492615

[33] Moon JS Hisata S Park MA DeNicola GM Ryter SW Nakahira K. Mtorc1-Induced HK1-Dependent Glycolysis Regulates Nlrp3 Inflammasome Activation. Cell Rep (2015) 12:102–15. 10.1016/j.celrep.2015.05.046 PMC4858438 26119735

[34] Rodríguez-Prados JC Través PG Cuenca J Rico D Aragonés J Martín–Sanz P. Substrate Fate in Activated Macrophages: A Comparison Between Innate, Classic, and Alternative Activation. J Immunol (2010) 185:605–14. 10.4049/jimmunol.0901698 20498354

[35] Boscá L Gonzá́lez-Ramos S Prieto P Ferná́ndez-Velasco M Mojena M Martín-Sanz P. Metabolic Signatures Linked to Macrophage Polarization: From Glucose Metabolism to Oxidative Phosphorylation. Biochem Soc Trans (2015) 43:740–4. 10.1042/BST20150107 26551722

[36] Jiang H Shi H Sun M Wang Y Meng Q Guo P. Pfkfb3-Driven Macrophage Glycolytic Metabolism is a Crucial Component of Innate Antiviral Defense. J Immunol (2016) 197:2880–90. 10.4049/jimmunol.1600474 27566823

[37] Palsson-McDermott EM Curtis AM Goel G Lauterbach MA Sheedy FJ Gleeson LE. Pyruvate Kinase M2 Regulates Hif-1α Activity and IL-1β Induction and Is a Critical Determinant of the Warburg Effect in LPS-Activated Macrophages. Cell Metab (2015) 21:65–80. 10.1016/j.cmet.2014.12.005 25565206 PMC5198835

[38] Xie M Yu Y Kang R Zhu S Yang L Zeng L. PKM2-Dependent Glycolysis Promotes NLRP3 and AIM2 Inflammasome Activation. Nat Commun (2016) 7:13280. 10.1038/ncomms13280 27779186 PMC5093342

[39] Freemerman AJ Johnson AR Sacks GN Milner JJ Kirk EL Troester MA. Metabolic Reprogramming of Macrophages: Glucose TRANSPORTER 1 (Glut1)-Mediated GLUCOSE Metabolism Drives A Proinflammatory Phenotype*. J Biol Chem (2014) 289:7884–96. 10.1074/jbc.M113.522037 PMC3953299 24492615

[40] Pavlou S Lindsay J Ingram R Xu H Chen M. Sustained High Glucose Exposure Sensitizes Macrophage Responses to Cytokine Stimuli But Reduces Their Phagocytic Activity. BMC Immunol (2018) 19:24. 10.1186/s12865-018-0261-0 29996768 PMC6042333

[41] Haschemi A Kosma P Gille L Evans CR Burant CF Starkl P. The Sedoheptulose Kinase CARKL Directs Macrophage Polarization Through Control of Glucose Metabolism. Cell Metab (2012) 15:813–26. 10.1016/j.cmet.2012.04.023 PMC3370649 22682222

[42] Semba H Takeda N Isagawa T Sugiura Y Honda K Wake M. Hif-1α-PDK1 Axis-Induced Active Glycolysis Plays an Essential Role in Macrophage Migratory Capacity. Nat Commun (2016) 7:11635. 10.1038/ncomms11635 27189088 PMC4873978

[43] van Uden P Kenneth NS Rocha S. Regulation of Hypoxia-Inducible factor-1alpha by NF-Kappab. Biochem J (2008) 412:477–84. 10.1042/BJ20080476 PMC2474706 18393939

[44] Rius J Guma M Schachtrup C Akassoglou K Zinkernagel AS Nizet V. NF-Kappab Links Innate Immunity to the Hypoxic Response Through Transcriptional Regulation of HIF-1alpha. Nature (2008) 453:807–11. 10.1038/nature06905 PMC2669289 18432192

[45] Chang YH Weng CL Lin KI Liu C Li L Herring LE. O-GlcNAcylation and its Role in the Immune System. J BioMed Sci (2020) 27:57. 10.1186/s12929-020-00648-9 32349769 PMC7189445

[46] Li T Li X Attri KS Liu C Li L Herring LE. O-Glcnac Transferase Links Glucose Metabolism to MAVS-Mediated Antiviral Innate Immunity. Cell Host Microbe (2018) 24:791–803 e796. 10.1016/j.chom.2018.11.001 30543776 PMC6296827

[47] Li X Gong W Wang H Li T Attri KS Lewis RE. O-Glcnac Transferase Suppresses Inflammation and Necroptosis by Targeting Receptor-Interacting Serine/Threonine-Protein Kinase 3. Immunity (2019) 50:576–590 e576. 10.1016/j.immuni.2019.01.007 30770249 PMC6426684

[48] Vats D Mukundan L Odegaard JI Zhang L Smith KL Morel CR. Oxidative Metabolism and PGC-1β Attenuate Macrophage-Mediated Inflammation. Cell Metab (2006) 4:13–24. 10.1016/j.cmet.2006.05.011 16814729 PMC1904486

[49] Namgaladze D Brune B. Fatty Acid Oxidation is Dispensable for Human Macrophage IL-4-induced Polarization. Biochim Biophys Acta (2014) 1841:1329–35. 10.1016/j.bbalip.2014.06.007 24960101

[50] Wu L Zhang X Zheng L Zhao H Yan G Zhang Q. Ripk3 Orchestrates Fatty Acid Metabolism in Tumor-Associated Macrophages and Hepatocarcinogenesis. Cancer Immunol Res (2020) 8:710–21. 10.1158/2326-6066.CIR-19-0261 32122992

[51] Wang F Zhang S Vuckovic I Jeon R Lerman A Folmes CD. Glycolytic Stimulation is Not a Requirement for M2 Macrophage Differentiation. Cell Metab (2018) 28:463–475.e464. 10.1016/j.cmet.2018.08.012 30184486 PMC6449248

[52] de–Brito--N Duncan–Moretti J da–Costa H Saldanha–Gama R Paula–Neto HA Dorighello G. Aerobic Glycolysis is a Metabolic Requirement to Maintain the M2-like Polarization of Tumor-Associated Macrophages. Biochim Biophys Acta Mol Cell Res (2020) 1867:118604. 10.1016/j.bbamcr.2019.118604 31760090

[53] Tan Z Xie N Cui H Moellering DR Abraham E Thannickal VJ. Pyruvate Dehydrogenase Kinase 1 Participates in Macrophage Polarization Via Regulating Glucose Metabolism. J Immunol (2015) 194:6082–9. 10.4049/jimmunol.1402469 PMC4458459 25964487

[54] Covarrubias AJ Aksoylar HI Yu J Snyder NW Worth AJ Iyer SS. Akt-mTORC1 Signaling Regulates Acly to Integrate Metabolic Input to Control of Macrophage Activation. eLife (2016) 5:e11612. 10.7554/eLife.11612 26894960 PMC4769166

[55] Huang SC Smith AM Everts B Colonna M Pearce EL Schilling JD. Metabolic Reprogramming Mediated by the Mtorc2-IRF4 Signaling Axis Is Essential for Macrophage Alternative Activation. Immunity (2016) 45:817–30. 10.1016/j.immuni.2016.09.016 PMC5535820 27760338

[56] Van den Bossche J Baardman J Otto NA van der Velden S Neele AE van den Berg SM. Mitochondrial Dysfunction Prevents Repolarization of Inflammatory Macrophages. Cell Rep (2016) 17:684–96. 10.1016/j.celrep.2016.09.008 27732846

[57] Yang Y Li X Luan HH Zhang B Zhang K Nam JH. OGT Suppresses S6K1-mediated Macrophage Inflammation and Metabolic Disturbance. Proc Natl Acad Sci U States Am (2020) 117:16616–25. 10.1073/pnas.1916121117 PMC7368321 32601203

[58] Zanin RF Braganhol E Bergamin LS Campesato LF Filho AZ Moreira JC. Differential Macrophage Activation Alters the Expression Profile of NTPDase and Ecto-5’-Nucleotidase. PloS One (2012) 7:e31205. 10.1371/journal.pone.0031205 22348056 PMC3278434

[59] Murphy PS Wang J Bhagwat SP Munger JC Janssen WJ Wright TW. CD73 Regulates Anti-Inflammatory Signaling Between Apoptotic Cells and Endotoxin-Conditioned Tissue Macrophages. Cell Death Differentiation (2017) 24:559–70. 10.1038/cdd.2016.159 PMC5344214 28060378

[60] Hartley GP Chow L Ammons DT Wheat WH Dow SW. Programmed Cell Death Ligand 1 (Pd-L1) Signaling Regulates Macrophage Proliferation and Activation. Cancer Immunol Res (2018) 6:1260–73. 10.1158/2326-6066.CIR-17-0537 30012633

[61] de Goede KE Driessen AJM Van den Bossche J. Metabolic Cancer-Macrophage Crosstalk in the Tumor Microenvironment. Biol (Basel) (2020) 9:380. 10.3390/biology9110380 PMC7694986 33171762

[62] Puthenveetil A Dubey S. Metabolic Reprograming of Tumor-Associated Macrophages. Ann Transl Med (2020) 8:1030. 10.21037/atm-20-2037 32953830 PMC7475460

[63] Wenes M Shang M Di Matteo M Goveia J Martín-Pérez R Serneels J. Macrophage Metabolism Controls Tumor Blood Vessel Morphogenesis and Metastasis. Cell Metab (2016) 24:701–15. 10.1016/j.cmet.2016.09.008 27773694

[64] Rodrigues Mantuano N Stanczak MA Oliveira IA Kirchhammer N Filardy AA Monaco G. Hyperglycemia Enhances Cancer Immune Evasion by Inducing Alternative Macrophage Polarization Through Increased O-Glcnacylation. Cancer Immunol Res (2020) 8:1262–72. 10.1158/2326-6066.CIR-19-0904 32819969

[65] Shapouri-Moghaddam A Mohammadian S Vazini H Taghadosi M Esmaeili SA Mardani F. Macrophage Plasticity, Polarization, and Function in Health and Disease. J Cell Physiol (2018) 233:6425–40. 10.1002/jcp.26429 PMC13482560 29319160

[66] Locati M Curtale G Mantovani. Diversity A. Mechanisms, and Significance of Macrophage Plasticity. Annu Rev Pathol (2020) 15:123–47. 10.1146/annurev-pathmechdis-012418-012718 PMC7176483 31530089

[67] Madden MZ Reinfeld BI Wolf MM Cohen AS Manning HC Rathmell WK. Nutrient Partitioning in the Tumor Microenvironment and FDG-PET Imaging. J Immunol (2020) 204:240.244.

[68] Liu D Chang C Lu N Wang X Lu Q Ren X. Comprehensive Proteomics Analysis Reveals Metabolic Reprogramming of Tumor-Associated Macrophages Stimulated by the Tumor Microenvironment. J Proteome Res (2017) 16:288–97. 10.1021/acs.jproteome.6b00604 27809537

[69] Penny HL Sieow JL Adriani G Yeap WH See Chi P San Luis B. Warburg Metabolism in Tumor-Conditioned Macrophages Promotes Metastasis in Human Pancreatic Ductal Adenocarcinoma. Oncoimmunology (2016) 5:e1191731. 10.1080/2162402X.2016.1191731 27622062 PMC5007961

[70] Arts RJ Plantinga TS Tuit S Ulas T Heinhuis B Tesselaar M. Transcriptional and Metabolic Reprogramming Induce an Inflammatory Phenotype in non-Medullary Thyroid Carcinoma-Induced Macrophages. Oncoimmunology (2016) 5:e1229725. 10.1080/2162402X.2016.1191731 28123869 PMC5213309

[71] Goetze K Walenta S Ksiazkiewicz M Kunz-Schughart LA Mueller-Klieser W. Lactate Enhances Motility of Tumor Cells and Inhibits Monocyte Migration and Cytokine Release. Int J Oncol (2011) 39:453–63. 10.3892/ijo.2011.1055 21617859

[72] Colegio OR Chu NQ Szabo AL Chu T Rhebergen AM Jairam V. Functional Polarization of Tumour-Associated Macrophages by Tumour-Derived Lactic Acid. Nature (2014) 513:559–63. 10.1038/nature13490 PMC4301845 25043024

[73] Zhang J Zhang Q Lou Y Fu J Chen J Wei J. Hypoxia-Inducible Factor-1α/Interleukin-1β Signaling Enhances Hepatoma Epithelial-Mesenchymal Transition Through Macrophages in a Hypoxic-Inflammatory Microenvironment. Hepatol (Baltimore Md.) (2018) 67:1872–89. 10.1002/hep.29681 29171040

[74] Jeong H Kim S Hong BJ Lee CJ Kim YE Bok S. Tumor-Associated Macrophages Enhance Tumor Hypoxia and Aerobic Glycolysis. Cancer Res (2019) 79:795–806. 10.1158/0008-5472.CAN-18-2545 30610087

[75] Kurahara H Shinchi H Mataki Y Maemura K Noma H Kubo F. Significance of M2-polarized Tumor-Associated Macrophage in Pancreatic Cancer. J Surg Res (2011) 167:e211–219. 10.1016/j.jss.2009.05.026 19765725

[76] Pan Y Lu F Fei Q Yu X Xiong P Yu X. Single-Cell RNA Sequencing Reveals Compartmental Remodeling of Tumor-Infiltrating Immune Cells Induced by anti-CD47 Targeting in Pancreatic Cancer. J Hematol Oncol (2019) 12:124. 10.1186/s13045-019-0822-6 31771616 PMC6880569

[77] Cheng H Wang Z Fu L Xu T. Macrophage Polarization in the Development and Progression of Ovarian Cancers: An Overview. Front Oncol (2019) 9:421. 10.3389/fonc.2019.00421 31192126 PMC6540821

[78] Arlauckas SP Garris CS Kohler RH Kitaoka M Cuccarese MF Yang KS. In Vivo Imaging Reveals a Tumor-Associated Macrophage-Mediated Resistance Pathway in anti-PD-1 Therapy. Sci Trans Med (2017) 9:eaal3604. 10.1126/scitranslmed.aal3604 PMC5734617 28490665

[79] Xue J Schmidt SV Sander J Draffehn A Krebs W Quester I. Transcriptome-Based Network Analysis Reveals a Spectrum Model of Human Macrophage Activation. Immunity (2014) 40:274–88. 10.1016/j.immuni.2014.01.006 PMC3991396 24530056

[80] Van Overmeire E Laoui D Keirsse J Van Ginderachter JA Sarukhan A. Mechanisms Driving Macrophage Diversity and Specialization in Distinct Tumor Microenvironments and Parallelisms With Other Tissues. Front Immunol (2014) 5:127. 10.3389/fimmu.2014.00127 24723924 PMC3972476

[81] Chen H Ye F Guo G. Revolutionizing Immunology With Single-Cell RNA Sequencing. Cell Mol Immunol (2019) 16:242–9. 10.1080/09546634.2019.1630701 PMC6460502 30796351

[82] Hartmann FJ Mrdjen D McCaffrey E Glass DR Greenwald NF Bharadwaj A. Single-Cell Metabolic Profiling of Human Cytotoxic T Cells. Nat Biotechnol (2021) 39:186–97. 10.1038/s41587-020-0651-8 PMC7878201 32868913

[83] Hartmann FJ Bendall SC. Immune Monitoring Using Mass Cytometry and Related High-Dimensional Imaging Approaches. Nat Rev Rheumatol (2020) 16:87–99. 10.1038/s41584-019-0338-z 31892734 PMC7232872

[84] Zhou B Magana L Hong Z Huang LS Chakraborty S Tsukasaki Y. The Angiocrine Rspondin3 Instructs Interstitial Macrophage Transition Via Metabolic-Epigenetic Reprogramming and Resolves Inflammatory Injury. Nat Immunol (2020) 21:1430–43. 10.1038/s41590-020-0764-8 PMC7815054 32839607

[85] Artyomov MN Van den Bossche J. Immunometabolism in the Single-Cell Era. Cell Metab (2020) 32:710–25. 10.1016/j.cmet.2020.09.013 PMC7660984 33027638

[86] Ruffell B Coussens LM. Macrophages and Therapeutic Resistance in Cancer. Cancer Cell (2015) 27:462–72. 10.1016/j.ccell.2015.02.015 PMC4400235 25858805

[87] Mantovani A Marchesi F Malesci A Laghi L Allavena P. Tumour-Associated Macrophages as Treatment Targets in Oncology. Nat Rev Clin Oncol (2017) 14:399–416. 10.1038/nrclinonc.2016.217 28117416 PMC5480600

[88] Bonapace L Coissieux MM Wyckoff J Mertz KD Varga Z Junt T. Cessation of CCL2 Inhibition Accelerates Breast Cancer Metastasis by Promoting Angiogenesis. Nature (2014) 515:130–3. 10.1038/nature13862 25337873

[89] Lee B Qiao L Kinney B Feng GS Shao J. Macrophage Depletion Disrupts Immune Balance and Energy Homeostasis. PloS One (2014) 9:e99575. 10.1371/journal.pone.0099575 24911652 PMC4049836

[90] Wu CL McNeill J Goon K Little D Kimmerling K Huebner J. Conditional Macrophage Depletion Increases Inflammation and Does Not Inhibit the Development of Osteoarthritis in Obese Macrophage Fas-Induced Apoptosis-Transgenic Mice. Arthritis Rheumatol (Hoboken N.J.) (2017) 69:1772–83. 10.1002/art.40161 PMC5611814 28544542

[91] Cassetta L Pollard JW. Targeting Macrophages: Therapeutic Approaches in Cancer. Nat Rev Drug Discovery (2018) 17:887–904. 10.1038/nrd.2018.169 30361552

[92] Yu Q Wang Y Dong L He Y Liu R Yang Q. Regulations of Glycolytic Activities on Macrophages Functions in Tumor and Infectious Inflammation. Front Cell Infect Microbiol (2020) 10:287. 10.3389/fcimb.2020.00287 32596169 PMC7303283

[93] Ashton TM McKenna WG Kunz-Schughart LA Higgins GS. Oxidative Phosphorylation as an Emerging Target in Cancer Therapy. Clin Cancer Res (2018) 24:2482–90. 10.1158/1078-0432.CCR-17-3070 29420223

[94] Bulle A Dekervel J Deschuttere L Nittner D Van Cutsem E Verslype C. Anti-Cancer Activity of Acriflavine as Metabolic Inhibitor of OXPHOS in Pancreas Cancer Xenografts. Onco Targets Ther (2020) 13:6907–16. 10.2147/OTT.S245134 PMC7369416 32764982

[95] Zhao Q Chu Z Zhu L Yang T Wang P Liu F. 2-Deoxy-D-Glucose Treatment Decreases Anti-inflammatory M2 Macrophage Polarization in Mice With Tumor and Allergic Airway Inflammation. Front Immunol (2017) 8:637. 10.3389/fimmu.2017.00637 28620389 PMC5451502

[96] Chaiyawat P Chokchaichamnankit D Lirdprapamongkol K Srisomsap C Svasti J Champattanachai V. Alteration of O-GlcNAcylation Affects Serine Phosphorylation and Regulates Gene Expression and Activity of Pyruvate Kinase M2 in Colorectal Cancer Cells. Oncol Rep (2015) 34:1933–42. 10.3892/or.2015.4178 26252736

[97] Wang Z Qin J Zhao J Li J Li D Popp M. Inflammatory IFIT3 Renders Chemotherapy Resistance by Regulating Post-Translational Modification of VDAC2 in Pancreatic Cancer. Theranostics (2020) 10:7178–92. 10.7150/thno.43093 PMC7330856 32641986

[98] Needham LA Davidson AH Bawden LJ Belfield A Bone EA Brotherton DH. Drug Targeting to Monocytes and Macrophages Using Esterase-Sensitive Chemical Motifs. J Pharmacol Exp Ther (2011) 339:132–42. 10.1124/jpet.111.183640 21778281

[99] Reichel D Tripathi M Perez JM. Biological Effects of Nanoparticles on Macrophage Polarization in the Tumor Microenvironment. Nanotheranostics (2019) 3:66–88. 10.7150/ntno.30052 30662824 PMC6328304

[100] Tavakoli S Zamora D Ullevig S Asmis R. Bioenergetic Profiles Diverge During Macrophage Polarization: Implications for the Interpretation of 18F-FDG PET Imaging of Atherosclerosis. J Nucl Med (2013) 54:1661–7. 10.2967/jnumed.112.119099 PMC4037161 23886729

[101] Lee SJ Thien Quach CH Jung KH Paik JY Lee JH Park JW. Oxidized Low-Density Lipoprotein Stimulates Macrophage 18F-FDG Uptake Via Hypoxia-Inducible Factor-1α Activation Through Nox2-dependent Reactive Oxygen Species Generation. J Nucl Med (2014) 55:1699–705. 10.2967/jnumed.114.139428 25214643

[102] Tavakoli S Short JD Downs K Nguyen HN Lai Y Zhang W. Differential Regulation of Macrophage Glucose Metabolism by Macrophage Colony-Stimulating Factor and Granulocyte-Macrophage Colony-Stimulating Factor: Implications for (18)F FDG Pet Imaging of Vessel Wall Inflammation. Radiology (2017) 283:87–97. 10.1148/radiol.2016160839 27849433 PMC5375627

